# Transition phase in acute respiratory distress syndrome: paving the way for our next major challenge

**DOI:** 10.1186/s13613-025-01566-5

**Published:** 2025-09-30

**Authors:** Anne-Fleur Haudebourg, Guillaume Carteaux

**Affiliations:** 1 Assistance Publique-Hôpitaux de Paris, CHU Henri Mondor-Albert Chenevier, Service de Médecine Intensive Réanimation, 51, Avenue du Maréchal de Lattre de Tassigny, 94010 Créteil Cedex, France; 2 Faculté de Santé, Groupe de Recherche Clinique CARMAS, Université Paris Est-Créteil, 94010 Créteil, France; 3 INSERM U955, Institut Mondor de Recherche Biomédicale, 94010 Créteil, France; 4 CHU Henri Mondor, Service de Médecine Intensive Réanimation, 1, rue Gustave Eiffel, 94010 Créteil Cedex, France

**To the editor,**

We thank Drs Xiong and Huang for their thoughtful comments [1] on our recent observational cohort describing the transition phase in moderate-to-severe acute respiratory distress syndrome (ARDS), a phase of mechanical ventilation that has been far less investigated than either the acute phase or the weaning process [2]. We agree that this “gray zone” deserves attention, as it may represent a turning point in the disease course. Epidemiological data on this underexplored phase were needed to identify key steps and associated risks, providing a foundation for future physiologic and interventional research. We identified two key steps during transition phase: neuromuscular blockade (NMBA) discontinuation and the switch from controlled to assisted ventilation.

The relatively high failure rates of these two steps (38% for NMBA withdrawal and 57% for pressure support ventilation (PSV) initiation), together with their association with worse outcomes, underscore the magnitude of this challenge. To our knowledge, our study is the first to report failure rates of NMBA weaning in ARDS. Moreover, our PSV failure rate aligns with recent findings (44% in series of 274 patients with mild-to-severe ARDS [3] and up to 67% in the recently published multicenter SWITCH cohort including nearly 7000 patients [4]), reinforcing the external validity of our observations and the need to unravel the underlying mechanisms.

We identified factors associated with failure in both transition steps. However, given the retrospective observational design, our aim was not to develop a predictive score but to report associations that may help generate hypotheses. While the link between failure and mortality might prompt caution, fear of failure should not justify unnecessarily prolonged controlled ventilation, which itself carries morbidity and mortality.

Regarding NMBA withdrawal, we acknowledge—as highlighted by Drs Xiong and Huang—that the discriminative accuracy of pH and PaO_2_/FiO_2_ in predicting the need for NMBA reinitiation was modest. This is why we explored thresholds with high specificity (pH < 7.30 or PaO_2_/FiO_2_ < 150 mmHg), aiming to help clinicians identify patients at particularly high risk of failure [2]. Whether transition should be avoided in such patients remains uncertain, and we fully recognize the limits of observational research in informing clinical recommendations. Still, these findings raise physiological questions about the need for monitoring and modulation of respiratory drive and effort in these patients. The potential U-shaped relationship between driving pressure and NMBA weaning failure is also intriguing. One could hypothesize that a high driving pressure reflects persistently impaired respiratory system compliance, which is usually associated with intense respiratory drive and, consequently, strong inspiratory effort at NMBA withdrawal, thereby exposing the patient to the risk of P-SILI. Conversely, a very low driving pressure may indicate a mismatch between the delivered tidal volume and the larger amount of aerated lung volume, insufficient to meet the patient’s demand, leading to air hunger and, ultimately, poor tolerance of NMBA discontinuation.

In our series, high tidal volume was associated with PSV failure. During PSV the tidal volume depends on respiratory mechanics, the level of assistance, and of patient effort. The absence of direct monitoring of effort is a limitation of our study, inherent to its retrospective design. However, respiratory mechanics and pressure support levels were similar between patients who succeeded and those who failed PSV, suggesting that the higher tidal volumes observed in the latter group were generated by greater respiratory drive and effort. Respiratory drive is influenced by multiple factors, including increased respiratory load due to reduced compliance or elevated resistance, but also impaired gas exchange, metabolic acidosis, systemic inflammation, anxiety, or pain. Furthermore, in certain circumstances, respiratory drive and inspiratory effort may be dissociated, adding further complexity to this phase and underscoring the need for physiological studies to elucidate the mechanisms underlying transition failure.

Since tools to measure and monitor respiratory drive and effort are now available not only for research but also for clinical practice [5], ongoing physiological studies will be valuable to clarify their role during the transition phase, to what extent they can be modulated, and how ventilator settings and monitoring can be individualized throughout this critical period.

In conclusion, we agree with Drs Xiong and Huang that future studies should integrate real-time effort-sensitive monitoring to refine management of this critical phase. Our study provides the first epidemiological evidence of the risks associated with NMBA withdrawal and PSV initiation in ARDS. Far from being the final word, it represents a necessary first step that helps pave the way for physiological hypotheses and the design of prospective trials aimed at improving the safety of transitions in ARDS.
